# Treatment of Pneumonitis

**Published:** 1874-04-15

**Authors:** A. Hermann

**Affiliations:** Pesth


### Translations.

### TREATMENT OF PNEUMONITIS.

By Dr. A. Hermann, of Pesth.

*Translated for* The Examiner, *from the Allgemeine Wiener Med. Zeitung, by* H. Gradle, M.D.

(Continued from Number VI.)

Having referred to the vast influence of age and the seat of the lesion on the result, some other points incident to the prognosis may be mentioned. A full, strong pulse is always a welcome sign; and, though the temperature reach 104° F., little anxiety need be experienced while the circulation continues vigorously; in fact, animal heat seems of little importance, in a prognostic view, since severe, even fatal cases, will often not advance beyond 102° F., while mild attacks may be accompanied by a high temperature, which is of no evil foreboding as long as the pulse does not become *small and frequent.* Constitution, temperament, "diathesis," appear of secondary importance; even previous diseases are not so detrimental as might be supposed; thus, of nine cases of pneumonitis supervening on phthisis, but one succumbed.

Approaching the more especial subject of these remarks, the treatment of the disease, Dr. Hermann at once declares that no remedy is known to abort pneumonitis; hence, but symptomatic indications can be filled. Experience has shown that subjective improvement in the patient's condition is synchronous with the abatement of fever, if the diminution of temperature and reduction in the frequency of the pulse constituted simultaneous events; hence, many have been the attempts, always unsuccessful, to anticipate nature in this respect, as advocates of which theory we need but mention Traube and Juergensen.

In his work on "Symptoms of Diseases of the Respiratory and Circulatory Systems," Traube says: "Thermometric observations of mine have shown several remedial measures possessing febrifuge properties. Digitalis has been mentioned. I need also refer to venesection, methodic refrigeration, and calomel. Their *modus operandi*, unknown to us, is of less import than the knowledge of their power. Furthermore, I have found that their influence on the temperature and the pulse *becomes more marked, according to their late application; at the height of the disease but little effect, if any, will sometimes follow their energetic use.*"

To the intelligent reader, these phrases simply confess the therapeutic inability of these methods to cut short inflammation of the lungs; and such an interpretation is but confirmed by the following sentences from another page: "Such experience induced me to consider the application of febrifuge remedies proper only after the acme of the affection had been passed; and since spontaneous defervescence occurs on a critical day, it seemed rational to employ these agents at the beginning of such critical days."

According to this mode of reasoning, *the happy period* for the *successful* physician to be called to the bedside, would be from the fifth to the seventh day, when either his remedies would effect a cure in twenty-four hours, where his competitor labored in vain well nigh a week, or death be inevitable after a week of previous "quackery."

As regards venesection, we can cite Traube for the following: "In twenty cases, depletion of six to eight ounces was resorted to, on the evening of the fourth or morning of the fifth day; and in every instance but one the crisis commenced on the fifth day, and was completed before the seventh."

The author affirms never to have practiced depletion; and still, of forty patients, of whom accurate thermometric record had been kept, no less than twenty-five had passed defervescence (Traube's crisis) before the close of the seventh day. Since, with but one exception, these patients were below fifty years of age, he inquires whether Traube's single case, in which venesection was a failure, had passed that period? The advice of the writer cited, though probably but little followed, is still to be deplored, since a man of his authority in other respects is not unlikely to thus mislead the inexperienced practitioner. If any advantage has been derived from it, it was to prove that, in pneumonitis, the loss of six to eight ounces of blood is not of great detriment.

Juergensen, in his previously quoted pamphlet, takes decided ground for the hydropathic treatment of the affection, on the theory that the cause of death in pneumonitis is found in cardiac insufficiency. Considering, as first effects of the inflammation, interference with the function of the lungs, and, secondarily, febrile excitement, Dr. Hermann cannot but agree with him; similarly, that neither condition will ordinarily account for a fatal termination individually, but only both united. Each morbid state, he continues, however, attacks life from its own point of application; but, together they may possess one in common, viz.: the heart. In argument, he urges that, while from the exudation in the lungs the resistance to the current of blood in the pulmonary circulation is increased, calling for augmentation of cardiac energy, less oxygen is absorbed to sustain the chemical processes necessary for further action of the heart; that, also, the retention of effete material more rapidly formed—the consequence of the fever—cannot but be detrimental to that organ; as the result of which injurious influences he finds it necessary to assume an early paralysis of the circulatory center. Death from annihilation of pulmonary function alone he opposes, on the ground that chronic pneumonitis, with obliteration of alveolar surface, or chronic pleuritic effusion, with complete compression of one lung, is not inconsistent with life for years. But here Dr. Hermann remarks that a rapid obstruction to respiration, such as sudden pneumo-thorax, is frequently fatal, when the same accident, gradually occurring, would permit the system to escape death by accommodation, or performance of the necessary processes through vicarious channels, which he clinically illustrates by the fatal termination of many cases of pneumonitis, resulting in sudden and extended infiltration of pulmonary cells, without high pyrexia.

Admitting that life closes with cardiac inactivity—for did the heart still beat, life would not be extinct—he denies this to be the direct cause of death; since, such a mere mechanical disturbance of the circulation, were it the moment on which cessation of life depends, would surely be productive of greater fatality than, happily, pneumonitis is attended with.

The point of attack from which the citadel of life is overcome, Dr. Hermann believes to be located in the medulla oblongata; and he sustains his theory by the following plausible arguments. Admitting that Juergensen's modes of paralysis of the heart exist, and possess such tendency, he doubts their power to accomplish, directly, ultimate annihilation of cardiac activity, since death is preceded by a hippocratic appearance, not by cyanosis, the sign of insufficiency of the heart; furthermore, life is not cut short by syncope, but by the gradual diminution of the (previously high) frequency of respiration, and the final cessation of breathing, indicative of paralysis of the vagus, and similar to the result of its section. Paralysis of motion is also observed in parts supplied by the other nerves originating in the medulla; the pharyngeal muscles, etc., for instance. At the same time, anæsthesia is evident in the sentient nerve-filaments leading to that center, hence no desire is experienced to relieve, by expectoration, the bronchial tubes of the accumulated mucous, till the quantity of fluid collected there may simulate the physical signs of pulmonary œdema. And though consciousness be not lost, relaxation of sphincters is of no rare occurrence. It has been said that, were inability of an encumbered heart to accomplish its augmented work, the starting-point of death, this unfortunate termination would be much more frequent than at present; for its conditions, especially their mechanical part, vary but little in different cases; but the little variation that does exist in the amount of oxygen absorbed, the quantity of effete material formed by the febrile tissue metamorphosis, and the proportion of such debris excreted by the various channels, is surely of much more influence on the delicate nerve-center, than on the comparatively energetic heart; besides, individual sensitiveness and powers of resistance are not the same in all persons; whence it is readily understood why death should select only special victims. And if we assume the last factor to be governed by the age, as statistics would warrant, the discrepancy in mortality at different periods of life finds a satisfactory explanation.

Refusing to accept Juergensen's theory, his treatment accordingly we need only discuss from an empirical standpoint, since its *modus operandi*, as stated by him, would only apply to his notions of the pathology of the disease. From his view of the cause of death, he recognizes two indications; 1. Prophylaxis of cardiac insufficiency; 2. Combating the paralysis that has begun. But, as he admits his inability to limit the pulmonary lesion, his advice is to attack the pyrexia, which he attemps by cold baths. Candidly, however, he confesses that theoretical objections to such procedures exist; for the spasm of peripheral arterioles, induced by the cold, adds to the resistance in the blood-channels, and increases the work the heart has to perform; whence collapse might be feared, though never yet experienced by his patients. To combat such result, he strongly advises the administration of stimulants before the bath, in amount proportionate to its low temperature and duration, laying especial stress on the utility of madeira or port-wine, in doses of one and a half f. ounces. "Besides, as a febrifuge, as well as a supporting stimulant, he recommends quinia, in the dose of one-half drachm every second day."

(*To be Continued.*)

